# The alignment of enzymatic steps reveals similar metabolic pathways and probable recruitment events in *Gammaproteobacteria*

**DOI:** 10.1186/s12864-015-2113-0

**Published:** 2015-11-17

**Authors:** Augusto Cesar Poot-Hernandez, Katya Rodriguez-Vazquez, Ernesto Perez-Rueda

**Affiliations:** Departamento de Microbiología Molecular, Instituto de Biotecnología, UNAM, Av. Universidad 2001, Cuernavaca, Morelos CP 62210 México; Departamento de Ingeniería de Sistemas Computacionales y Automatización, Instituto de Investigaciones en Matemáticas Aplicadas y en Sistemas, UNAM, Ciudad Universitaria, CP 04510 México D.F. México

**Keywords:** Metabolism, Pathway alignment, Gammaproteobacteria, Enzyme commission number

## Abstract

**Background:**

It is generally accepted that gene duplication followed by functional divergence is one of the main sources of metabolic diversity. In this regard, there is an increasing interest in the development of methods that allow the systematic identification of these evolutionary events in metabolism. Here, we used a method not based on biomolecular sequence analysis to compare and identify common and variable routes in the metabolism of 40 *Gammaproteobacteria* species.

**Method:**

The metabolic maps deposited in the KEGG database were transformed into linear Enzymatic Step Sequences (ESS) by using the breadth-first search algorithm. These ESS represent subsequent enzymes linked to each other, where their catalytic activities are encoded in the Enzyme Commission numbers. The ESS were compared in an all-against-all (pairwise comparisons) approach by using a dynamic programming algorithm, leaving only a set of significant pairs.

**Results and conclusion:**

From these comparisons, we identified a set of functionally conserved enzymatic steps in different metabolic maps, in which cell wall components and fatty acid and lysine biosynthesis were included. In addition, we found that pathways associated with biosynthesis share a higher proportion of similar ESS than degradation pathways and secondary metabolism pathways. Also, maps associated with the metabolism of similar compounds contain a high proportion of similar ESS, such as those maps from nucleotide metabolism pathways, in particular the inosine monophosphate pathway. Furthermore, diverse ESS associated with the low part of the glycolysis pathway were identified as functionally similar to multiple metabolic pathways. In summary, our comparisons may help to identify similar reactions in different metabolic pathways and could reinforce the *patchwork model* in the evolution of metabolism in *Gammaproteobacteria*.

**Electronic supplementary material:**

The online version of this article (doi:10.1186/s12864-015-2113-0) contains supplementary material, which is available to authorized users.

## Background

The study of the evolution of metabolism is central to understanding the adaptive processes of cellular life, the emergence of high levels of organization (multicellularity), and the diversity and complexity of the living world [1, 2]. At present, the large-scale information derived from genomic and proteomic studies has allowed the development of databases devoted to organizing the metabolic processes, such as the KEGG [3] and MetaCyc [4]. The information contained in these databases can be used to generate an integrative perspective of cellular functioning.

Metabolism can be considered one of the most ancient biological networks, where the nodes represent substrates and/or enzymes and the edges represent the relationships among them. From this perspective, the study of metabolic networks has focused on describing topological properties and has showed the existence of a structured network architecture [5–7]. Another relevant feature of metabolic networks is their modularity [8, 9], where each module is a discrete entity of elementary components (enzymes and substrates) that performs a certain task, separable from the functions of other modules. The elements of each module are related to each other and may be subjected to the same evolutionary process, such as amino acid biosynthesis, where a high rate of duplication events has been identified [10]. In this regard, metabolic pathways exhibit high retention of duplicates within functional modules and a preferential biochemical coupling of reactions. This retention of duplicates may result from the biochemical rules governing substrate-enzyme-product relationships [11–13].

In this work, we ask whether there are groups of similar reactions in different or in the same metabolic pathways, which might suggest a transfer of enzymatic activities, and whether these groups can be used to define common and variable metabolic pathways in 40 organisms belonging to the *Gammaproteobacteria* division. *Gammaproteobacteria* are excellent models to consider because they contain a large diversity of species [14], such as the bacterium *Escherichia coli*K-12, for which a large number of molecular and functional mechanisms have been elucidated. In addition, *Gammaproteobacteria* include organisms widely distributed throughout diverse environments, such as the endocommensal bacterium *Ruthia magnifica* [15], obligate endosymbionts *Baumannia* sp. and *Buchnera* sp., photoautotrophs such as *Halorhodospira halophile* [16], and mammal pathogens, such as *Yersinia* spp. and *Vibrio* spp., among others [17, 18].

To this end, we implemented a general strategy that considers the transformation of the metabolic maps deposited in the KEGG database into linear Enzymatic Step Sequences (ESS) and their posterior comparison with a dynamic programming sequence alignment algorithm. From these comparisons, we show that maps associated with the metabolism of similar compounds also contain a high proportion of similar ESS. In addition, we evaluate the possible contribution of two ancient pathways, glycolysis and IMP, to the metabolic pathways growth. Finally, we consider that our comparisons may provide clues reinforcing the *patchwork model* in the evolution of metabolism in *Gammaproteobacteria*.

## Results

### Construction and comparison of ESS

**Fig. 1:**
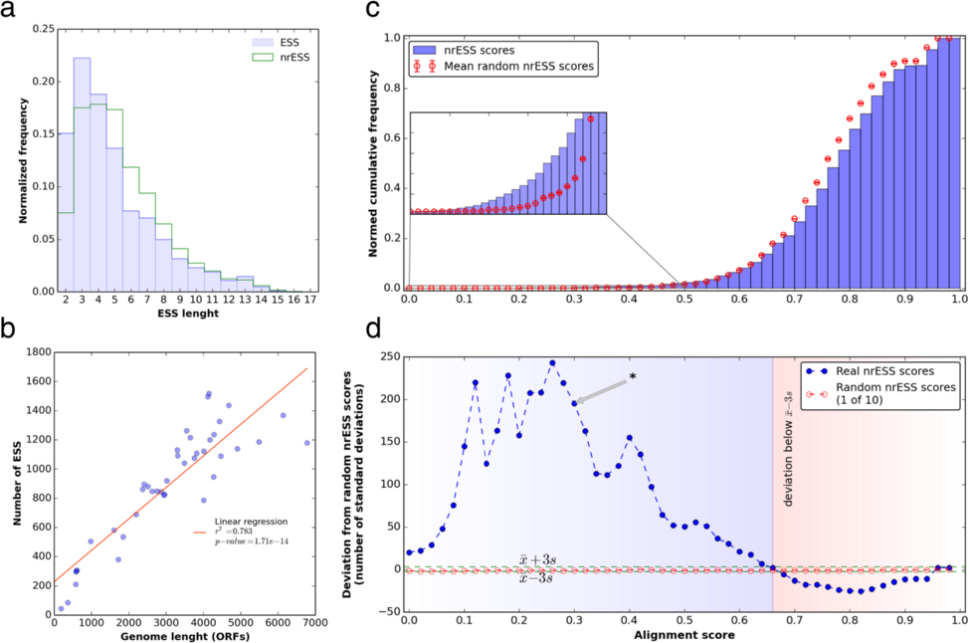
Construction and alignment of Enzymatic Step Sequences (ESS). **a** Histogram of the distribution of ESS and nrESS according to their lengths. **b** Number of total ESS constructed per organism. The *x*-axis represents the genome size according to the number of ORFs, and the *y*-axis corresponds to the total number of ESS generated using the strategy presented in the text. Each point corresponds to one organism. **c** Cumulative histogram of the nrESS pairwise alignment scores. Blue bars represent the real nrESS scores; red points represent the mean distribution ± SD from random ESS. The SD were so small that they are not visible in the plot. The inset is an amplification of the lower portion of the plot and includes the scores from 0 to 0.5. **d** Deviation of the real nrESS score histogram relative to the mean ± SD of the random datasets. The blue dots correspond to the real data dispersion, and red dots correspond to single random database dispersions. Dotted green lines represent 3 SD from the mean of random histograms. The asterisk denotes the dispersion at 0.3. This score was used as the similarity threshold

In order to evaluate the commonalities and differences in the metabolism of organisms belonging to the *Gammaproteobacteria* division, a collection of ESS was generated from their corresponding metabolic maps. In this regard, an ESS was defined as a linear collection of consecutive enzymatic reactions from a given substrate to a given product, in a similar way as a previously proposed definition of metabolic pathways [19, 20]. To do this, the breadth-first search (BFS) algorithm was used, as we describe in Material and Methods. This algorithm allows the systematic fragmentation of metabolic pathways for the alignment analysis, and it has been used to identify the shortest pathway between compounds in metabolic networks [21]. Therefore, each ESS was reconstructed following subsequent reactions in each metabolic map. The enzymes related to each reaction were represented by using the first three levels of the Enzyme Commission (EC) number classification to describe their general type of chemical reaction, as it was previously suggested [22]. In total, 2973 KEGG maps from 40 species were analyzed, of which 2284 generate at least one sequence. The remaining 689 maps did not generate any sequence because they contain few enzymes, contain ramification pathways or describe transport mechanisms, or the enzymes do not have connections among them (Additional file 1: Table S1). Therefore, the length distribution of the total 36,621 constructed ESS ranged from 2 to 17 enzymatic steps, with a mean length of 5 and a mode equal to 3 (Fig. 1a). In addition, we found a correlation between the genome size (in open reading frames, or ORFs) and the number of ESS, as large genomes generated more ESS (*r*^2^ = 0.78, *p* = 1.7x10^-14^) than small genomes (Fig. 1b). In a similar way, the number of ESS generated per metabolic map also depended on the number of ORFs associated with each map (*r*^2^ = 0.581, *p* ≈ 0) (Additional file 2: Figure S1). These results suggest that the number of ESS reflects to some extent the increased complexity in metabolism as a function of the number of proteins contained in an organism.

A natural observation that emerged from these sequences concerns their redundancy, i.e., identical ESS derived from different organisms. To reduce this redundancy and to facilitate the subsequent analysis, identical sequences were identified and excluded, leaving a representative of them and defining the non redundant ESS (nrESS) dataset. From this filtering, 7970 different nrESS were considered for posterior analyses. The nrESS length histogram was similar to that for the complete set of ESS, with a mean length of 5.4 and a mode equal to 4 (Fig. 1a). In this report, we refer only to the nrESS.

In a second step, the nrESS were compared by using the dynamic programming Needleman and Wunsh (NW) algorithm in an all-against-all strategy. The alignment generated by this algorithm was evaluated by using an entropy based normalized function that yields values in the interval from 0 to 1. Hence, values close to 0 mean less entropy and more homogeneous columns in the alignment, reflecting more similar nrESS. Conversely, values close to 1 reflect dissimilar nrESS.

From these comparisons, we found that the distribution of the scores resembled an extreme value Gumbel distribution (Additional file 2: Figure S2), with the highest proportion of the scores close to 1, i.e., the major proportion of alignments occurs between dissimilar nrESS. To evaluate the statistical significance of all comparisons, 10 random databases were generated by shuffling the real nrESS, maintaining the EC composition and length sizes. The random databases were analyzed in the same all-against-all fashion, and the resulting scores were compared against real alignment scores. In Fig. 1c we show the cumulative histogram of the alignment scores of the real and random datasets. Based on this analysis, scores close to 0 are overrepresented in real data compared to random nrESS. To evaluate this overrepresentation, the deviation of the real dataset relative to the mean ± standard deviation of the 10 random datasets was calculated (Fig. 1d). According to these data, the real and random scores intersect at 0.65, suggesting that this value is the limit to identify distant similarities; therefore, an alignment with a score of ≈ 0.65 may be considered clearly random. Based on this information, a significant alignment threshold was established to analyze the most of the nrESS, with not compromising the statistical relevance. Therefore, a score of ≤0.3 was established as threshold. This value represents the higher dispersion (>195 SD) of the random data (Fig. 1d, asterisk) with the lowest loss of nrESS, i.e., more than 99 % of the real nrESS were included (Additional file 2: Figure S3). This threshold also corresponds to 0.26 % of all nrESS alignments (81,520 of 31,756,465) and includes 7907 out of 7970 nrESS. In contrast, from the alignments associated with the 10 random databases (31,756,465 for each dataset), only 0.04 ± 0.001 % (13,827 ± 308) of the total alignments exhibited a threshold of ≤0.3. These results show that our method can be used to identify similar nrESS with significant scores, excluding the possibility of finding such similar nrESS by random chance. Here, we report information concerning our comparisons of these nrESS related to metabolism in diverse bacterial organisms.

### Pairwise alignments of nrESS identify a core of common metabolic pathways in *Gammaproteobacteria*

**Fig. 2:**
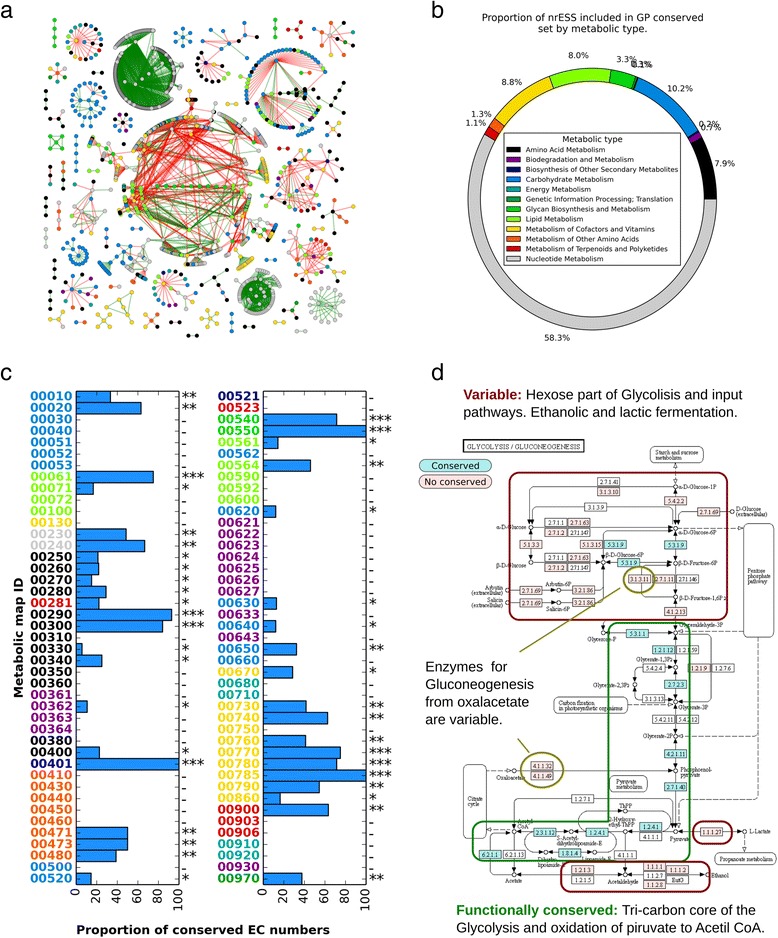
Functional conserved and variable nrESS in *Gammaproteobacteria*. **a** Graph representation of the relationships between the set of conserved ESS in *Gammaproteobacteria*. The nodes represent the nrESS and the edges show the alignments among them. Green edges are alignments between the same metabolic map, and the red ones represent alignments between different metabolic maps. The nodes are colored according to the metabolism type, as indicated. The alignments between ESS from the same map were selected as the Metabolic Map Functional Conserved Dataset (MMFCD). **b** Proportion of ESS from each metabolic type included in the MMFCD. **c** Proportion of EC numbers conserved in the alignments of the MMFCD for each metabolic map. The metabolic maps are represented by the KEGG map ID, and the colors indicate the metabolic type. The metabolic maps were classified according to the proportion of functional conserved EC numbers, as follows: conserved (***), more than 70 % of EC numbers conserved; moderately conserved (**), between 30 and 69 % of numbers conserved; barely conserved (*), between 1 and 30 % of numbers conserved; non conserved (-), 0 % of numbers conserved. **d** Functional conservation of the glycolysis/gluconeogenesis metabolic map in *Gammaproteobacteria*. The map represents the functional mapping of conserved nrESS (3 levels of EC classification) in the alignments of the MMFCD (cyan). In addition, the nrESS present in at least one of the species analyzed but not in the conserved set are shown (pink). The white steps represent enzymes not present in any of the species analyzed. In boxes and circles are represented the conserved and variable regions in the metabolic map. See text for details

In order to evaluate the similarity of the metabolic maps in *Gammaproteobacteria* and whether there is a group of *functionally conserved* pathways in these organisms, a set of similar nrESS was defined. In this context, the term *functionally conserved* refers to the identification of similar nrESS that may be common to *Gammaproteobacteria*. Two nrESS were considered as *functionally conserved* if their alignment had a score below the threshold (≤0.3) and, in conjunction, they were present in more than 75 % of the species analyzed. Based on this definition, the set included 1484 sequences from 74 different metabolic maps, with 69 % of the total alignments corresponding to alignments between the same metabolic maps (1805 of 2633), whereas 31 % corresponded to alignments between different metabolic maps (Fig. 2a).

To assess the nrESS similarity of each metabolic map as an indicator of functional conservation, we used the alignments that occurred within them (green edges in Fig. 2a), and we named this dataset the Metabolic Map Functional Conserved Dataset (MMFCD). The proportion of each metabolic type represented in this dataset is shown in Fig. 2b, and corresponds primarily to nrESS of the metabolism of nucleotides, followed by the metabolism of carbohydrates, cofactors and vitamins, amino acids, and lipids. In contrast, the pathways for xenobiotic biodegradation and metabolism, biosynthesis and other secondary metabolism, metabolism of other amino acids, and metabolism of terpenoids and poliketides, among others, represent less than 5 % of the total nrESS included in the dataset. From these alignments, we mapped the position of the highly similar nrESS in the corresponding metabolic map to determine the proportion of the *functionally conserved* EC numbers in relation to the total EC numbers present in *Gammaproteobacteria* (Fig. 2c). Using this information, we classified the metabolic maps into four groups: 1) maps with more than 70 % of the EC numbers identical, i.e. highly *functionally conserved*; 2) moderately *functionally conserved* maps, with percentages between 30 % and 69 %; 3) barely *functionally conserved*, i.e., those maps with percentages between 1 % and 29 %; finally, 4) variable maps, i.e., with 0 % EC classified as *functional conserved*. From these data, less than one-third of the analyzed maps (24 of 86) were classified as highly or moderately *functionally conserved*, while more than two-thirds were considered as barely *functionally conserved* or variable. All these data showed that more than half of the metabolic maps analyzed did not exhibit common nrESS in *Gammaproteobacteria* and, by consequence, may be considered variable, suggesting a high variability in the metabolism of this bacterial division.

In detail, maps classified as highly *functionally conserved* are related to important processes, like the pathways for fatty acid biosynthesis (map00061), metabolism of some amino acids (00290 for valine, leucine, and isoleucine biosynthesis; 00300 for lysine biosynthesis), components of the cell wall (00540 for lipopolysaccharide biosynthesis; 00550 for peptidoglycan biosynthesis), metabolism of some cofactors (00770 for pantothenate and CoA biosynthesis; 00780 for biotin metabolism; 00785 for lipoic acid metabolism), and novobiocin biosynthesis (00401). These functional similarity also correlate with the fact that amino acid metabolism pathways for valine, leucine, isoleucine, and lysine have been identified as pathways with diverse duplicated genes in the three cellular domains of life [10, 23].

The second group includes those maps defined as moderately *functionally conserved*. In this category were included the pathways for metabolism of purines (00230) and pyrimidines (00240), glycolysis/gluconeogenesis (00010), the citrate cycle (00020), metabolism of glycerophospholipids (00564), terpenoids backbone (00900), and some cofactors, like riboflavin (00740), nicotinamide (00760), folate (00790), and thiamine (00730). It is interesting that the central part of glycolysis (00010), the Embden-Meyerhof pathway, is partially conserved among *Gammaproteobacteria* (Fig. 2d), whereas the core pathway that comprises the tricarbon compounds is widely *functionally conserved* among the analyzed organisms, including the oxidation of pyruvate to acetyl CoA. In the hexose section, the enzymatic steps catalyzed by 6-phosphofructokinase (EC2.7.1.11) and fructose biphosphate aldolase (EC4.1.2.13) are considered variable. A similar result was observed with the glycolysis input, where the mechanisms by which the hexoses enter the pathway are variable. In addition, the enzymatic steps to transform pyruvate to lactate and the ethanolic fermentation from acetate are also variable. In a similar way, gluconeogenesis from oxaloacetate is partially *functionally conserved* in *Gammaproteobacteria*, where the enzymes allowing the input from the oxaloacetate (phosphoenol pyruvate carboxykinase, EC4.1.1.49 and 4.1.1.32) and the enzyme that dephosphorylates fructose 1,6-bisphosphate to fructose 6-phosphate (fructose biphosphatase, EC3.1.3.11) are considered variable. These results are congruent with those from a previous study, where it was concluded that glycolysis is a plastic pathway and that the lower part of the glycolysis pathway is the more conserved section among the three cellular domains [24].

**Fig. 3:**
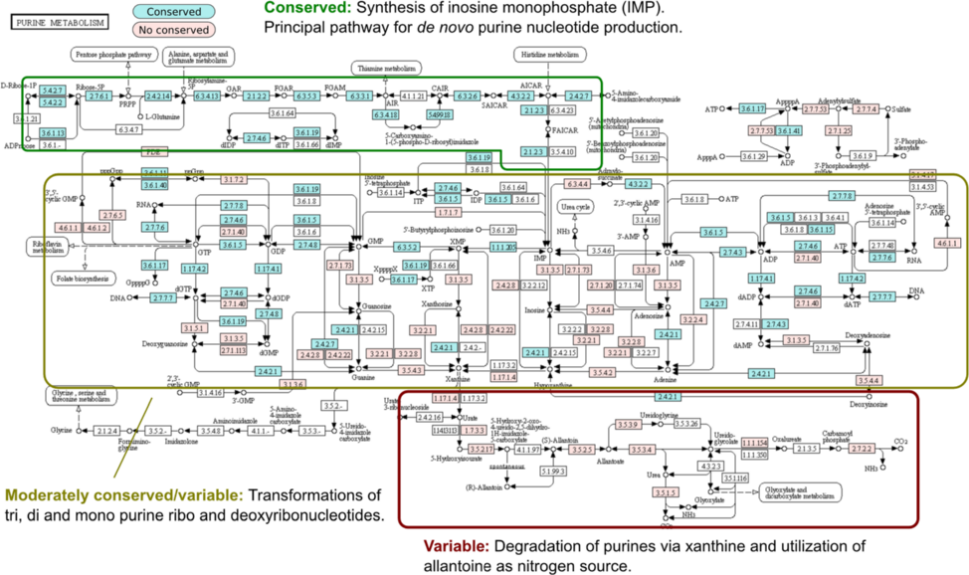
Functional conservation of purine metabolism in *Gammaproteobacteria*. The map shows the mapping of functional conserved enzymatic steps (three levels of EC classification) in the alignments of the metabolic map conserved dataset (cyan). The enzymatic steps present in at least one of the species analyzed but not in the conserved set are shown (pink). The white steps represent enzymes do not present in *Gammaproteobacteria*. In boxes are represented the conserved and variable regions in the metabolic map

Another example is the case of purine (00230) and pyrimidine (00240) metabolism. In general, both metabolic maps show *functionally conserved* reactions that converge the synthesis of mono-, di-, and triphosphate ribonucleotides and deoxyribonucleotides. In the case of purine metabolism (Fig. 3), the biosynthetic pathway for the main precursor to the synthesis of purine nucleotides [25], inosine monophosphate (IMP) is completely conserved in *Gammaproteobacteria* (KEGG module M00048). Congruently, the classical synthesis of ATP (module M00049) and GTP (module M00050) from IMP is also *functionally* conserved, although there are many variable enzymatic steps that catalyze the production of nucleosides and nitrogenous bases. Finally, the pathways for degradation of purines via xanthine and allantoate utilization are variable in *Gammaproteobacteria*. Therefore, in purine metabolism we observed a general *functional conservation* of synthetic pathways and a general *non-functional conservation* of degradation pathways. These results, in conjunction with recent data suggesting that nucleotide metabolism is highly conserved across all the organisms [26], reinforce the notion that purine biosynthesis is one of the more ancient metabolic pathways [1, 27].

A similar conservation pattern is observed in other metabolic maps classified as moderately *functionally conserved*, such as the pathway for glycerophospholipid metabolism (00564). We found that the biosynthetic pathways to CDP-diacylglycerol and then to phosphatidyl glycerol, phosphatidyl serine, and phosphatidyl ethanolamine are conserved, while the biosynthetic pathway to phosphatidyl choline and the degradation pathways are variable. A similar result arises for the biosynthesis of cofactors like thiamine-diphosphate (map 00730), riboflavin (map 00740), NAD^+^ and NADP^+^ (map 00760), and tetrahydrofolate (map 00790). In conjunction, it is possible to deduce a *functional conservation* pattern for *Gammaproteobacteria*, where some metabolic maps contain a biosynthesis-related core of similar enzymatic steps, and some variable steps that include the degradation of various compounds. These variable or dispensable steps may represent possible alternative pathways in different organisms and/or in different ecological niches, as has been previously suggested [10, 28].

The group of metabolic maps classified as barely *functionally conserved* includes important processes, such as amino acid metabolism, fatty acid degradation (beta-oxidation), and glycerolipid metabolism. In this context, we identified many variable reactions in the map that describes alanine, aspartate, and glutamate metabolism (map 00250), suggesting the existence of alternative pathways to produce these compounds. In this regard, there are three possible enzymes that catalyze the conversion of L-glutamate to L-glutamine: one of them by a ligation reaction (glutamine synthetase, EC6.3.1.2) and two by reversible hydrolysis (glutaminase, EC3.5.1.2, and L-glutamine (L-asparagine) amidohydrolase, EC3.5.1.38). In particular, the L-glutamine (L-asparagine) amidohydrolase also catalyzes the deamination of asparagine to aspartate. This finding suggests more flexible networks for the production of amino acids and reinforces the notion of various alternative enzymes for the production of amino acids [10]. A similar observation arises for cysteine and methionine metabolism (map 00270), for which alternative pathways were also identified. For example, the pathway to produce methionine from aspartate (module M00017) is not completely conserved in *Gammaproteobacteria*; nevertheless, there are some alternative enzymes that may work as alternative paths for the synthesis of methionine. Interestingly, some of these alternative enzymes were identified as functionally conserved in this work, suggesting not only the absence of a conserved canonical route but also important alternative enzymatic steps.

Finally, the *variable* maps include a high diversity of metabolisms types. Some of them contain few or fragmented enzymatic steps present in at least one *Gammaproteobacteria* species, suggesting the absence of those metabolic maps in this clade. However, other maps contain many enzymes present in *Gammaproteobacteria*; such as those for seleno compound metabolism, galactose metabolism, pentose phosphate and pentose metabolism, and glucuronate metabolism, among others. In general, the maps classified in this category represent pathways for degradation of uncommon compounds (xenobiotics) or for alternative carbon sources (carbohydrate metabolism). Altogether, these observations in addition to supporting the previously proposed idea concerning the reduced conservation of degradation related pathways; reinforce the notion of differential enzyme recruitment across the clade. Also, our results support the proposed preponderance of central carbon and anabolic pathways in the evolution of metabolism [2, 8, 27, 29].

In summary, all these data allow the identification of a *core* of similar enzymatic steps in *Gammaproteobacteria*. This *core* includes primarily reactions of the central carbon metabolism (low part of glycolysis and tricarboxylic acid cycle), and the biosynthetic pathways for nucleotides, cofactors and some amino acids. In addition this *core* is complemented with a set of variable pathways that primarily includes degradation pathways for carbohydrates, amino acids and xenobiotics that may be essential to the particular life style of each organism.

The complete set of functional conservation of metabolic maps in *Gammaproteobacteria* is available as KEGG weblinks in Additional file 3: File S2.

### Metabolic maps that convert similar compounds also share similar nrESS

**Fig. 4:**
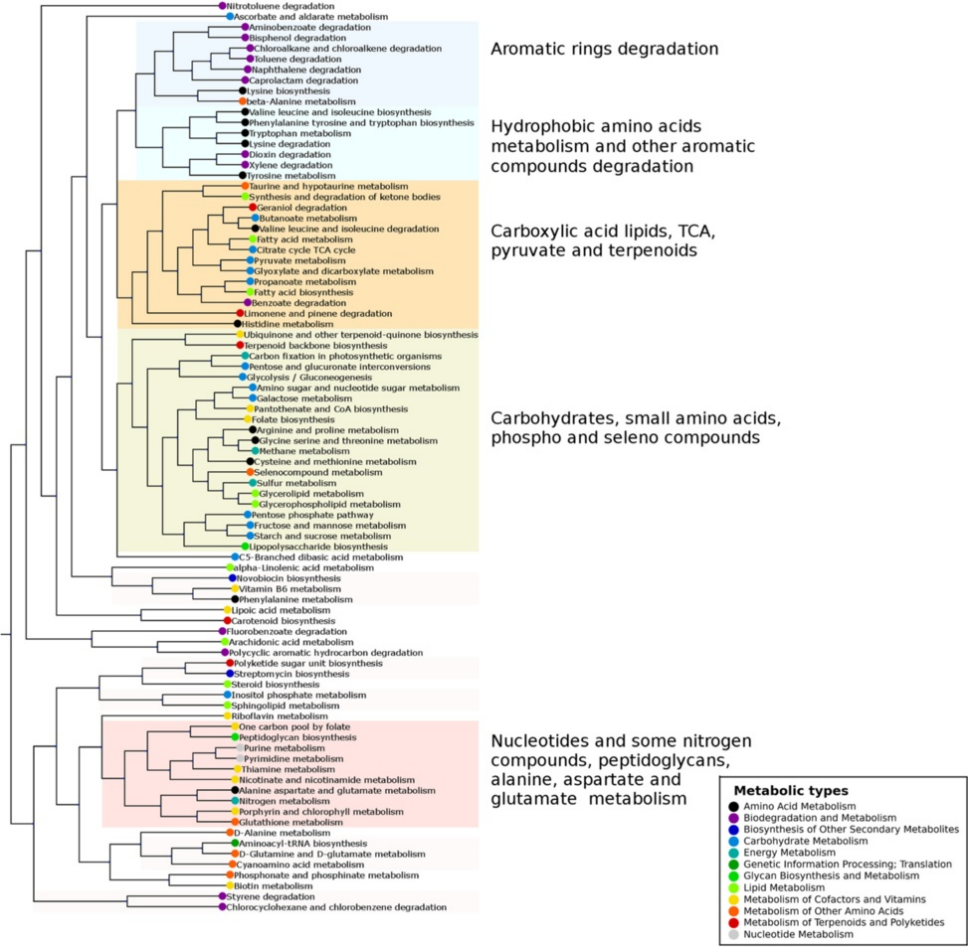
The metabolic map that includes similar compounds exhibits similar ESS. The hierarchical clustering was constructed by comparing the normalized counts of shared pairwise nrESS alignments, using the Spearman rank correlation and average linkage method. A cutoff of 0.46 of the total length of the clustering was used to define the number of clusters. The major and minor (pale pink) clusters are shown, and their corresponding KEGG metabolic types are indicated

In this section, we asked whether the similarities between nrESS might help to identify those metabolic maps that convert similar compounds and uncover explicitly the functional relations between metabolic maps. In this regard, we explored the general similarities between metabolic maps identified by nrESS comparisons. To do so, the total number of shared alignments between each pair of metabolic maps was calculated, considering only those alignments with scores of ≤0.3. The counts of the shared alignments were normalized to the total number of alignments for each map and used as similarity vectors in a hierarchical clustering analysis with the Spearman rank correlation as similarity measure (Fig. 4). Considering a cutoff of 0.46 of the total length of the clustering tree, we defined a total of 24 different metabolic map clusters. Similar results were obtained using Kendal rank correlation and self-organizing maps. This analysis showed 5 clusters that included more than 3 metabolic maps, 5 clusters that included 2 or 3 maps, and 14 maps that were considered singletons. The first of the major clusters included metabolic maps related to the degradation of aromatic compounds, such as amino benzoate, bisphenol, toluene, and naphthalene, among others. The second major cluster included hydrophobic amino acid metabolism (e.g., for tryptophan, phenylalanine, and tyrosine) and aromatic compound degradation (e.g., of xylene and dioxins). The third cluster contained carboxylic acid (fatty acids and butanoate), long aliphatic chain lipids (e.g., limonene and geraniol), valine, leucine, and isoleucine degradation, and pyruvate and the TCA cycle maps. This result suggests a functional similarity between pyruvate and the TCA cycle and the synthesis of aliphatic chain hydrophobic carboxylic acids. The fourth cluster includes the metabolism of carbohydrates, carbon fixation, metabolism of some cofactors (CoA and folate), terpenoids, glycerolipids, and sulfur (including methionine and cysteine) and seleno compounds. Finally, the fifth cluster contains the metabolism of nucleotides, peptidoglycans, nitrogen metabolism, and other nitrogen-containing cofactors, like hiamine and nicotinamide. In summary, we identified a trend where metabolic maps describing the transformations of chemically similar molecules also contained similar nrESS, probably as consequence of enzymatic recruitment.

### Similar nrESS suggest that enzyme recruitment is a frequent event in metabolism of *Gammaproteobacteria*

**Fig. 5:**
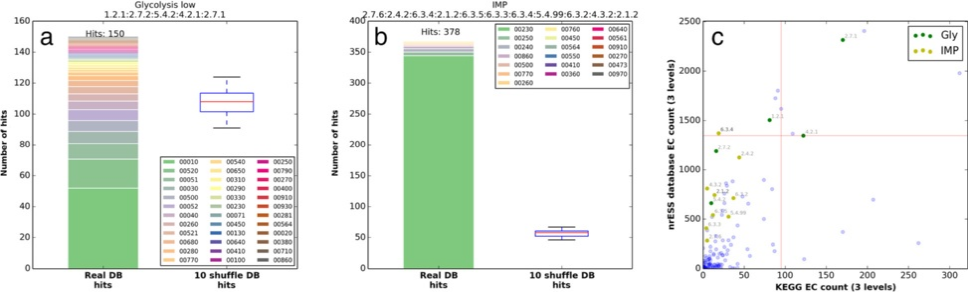
The similarities between nrESS suggest enzyme recruitment. Two nrESS representing the lower part of the glycolysis pathway (**a**) and the Inosine Monophosphate (IMP) pathway (**b**) were compared against the nrESS database (bar) and 10 random databases (boxplot). The number of significant hits (score of ≤0.3) was plotted. The color code indicates the proportion of hits corresponding to each metabolic map. **c** The relation between the number of EC numbers sharing the first 3 levels of classification in the KEGG database versus the number of occurrences of each EC number in the nrESS database

Based on the previous sections, we ask if *functionally conserved* pathways can be used to identify the possible recruitment patterns in the metabolism of *Gammaproteobacteria*. In this context, the corresponding nrESS of the lower part of the glycolysis pathway and the IMP pathway for *de novo* synthesis of purines were used to scan the complete nrESS dataset. Both pathways are considered ancient [24, 30]. We used the NW algorithm with a score threshold of 0.3. To determine the significance of the alignments we also scanned the random nrESS. From these comparisons, we found that the numbers of significant matches for the lower part of the glycolysis pathway (Fig. 5a) and the IMP pathway (Fig. 5b) were greater than those expected by chance; however, the difference relative to the random databases was greater for IMP than for glycolysis. In addition, we determined that although the raw number of hits was greater for IMP (381) than for glycolysis (148), the number of alignments with other maps was greater for glycolysis than for IMP (37 versus 19 different metabolic maps). The relatively small difference in the number of significant matches obtained for glycolysis compared to the random databases may be explained in part by the number of occurrences of its constituent EC numbers in the nrESS database and in the KEGG database (Fig. 5c). The EC numbers 1.2.1 (oxidoreductases acting on the aldehyde or oxo group of donors and with NAD^+^ or NADP^+^ as acceptor), 2.7.1 (phosphotransferases with an alcohol group as acceptor), and 4.2.1 (carbon-oxygen hydrolyases) are within the top 10 in nrESS database abundance. This result shows a broad similarity of the lower part of the glycolysis pathway with many other metabolic processes and suggests similar catalytic processes are used to transform some compounds in different metabolic maps. In turn, this observation suggests an outstanding proportion of enzyme recruitment events from glycolysis to other metabolic pathways and may reflect the utilization of similar products generated for similar reactions in different metabolic maps. On the other hand, the major number of hits for the IMP pathway corresponds to alignments within the same map, suggesting that this pathway has increased its size by duplication and recruitment of its own enzymes. Although both metabolic pathways may be considered ancient and were classified as functional conserved in this study, the patterns of functional similarities are different and may reflect the constraints of enzyme recruitment and the ubiquity of some types of compounds.

## Discussion and conclusions

In this work we used a simple workflow for the comparative study of metabolism through the alignment of linear sequences of ESS. The metabolic maps stored in KEGG were transformed into linear ESS by using an exhaustive and well-defined graph search algorithm. Then, the ESS were compared to identify the commonalities and differences between them. This approach allows the identification of similarities at the Enzymatic Step Sequences (ESS) level in a set of metabolic pathways. In this regard, the use of the functional information of the enzyme activity rather than the (protein and DNA) sequence information suggest that metabolism comprises a complex and dynamic network that may have different proteins to achieve the same or similar function.

Diverse methods for the alignment of biological networks have been suggested, such as protein-protein interaction networks (for some examples see references [31–33]) and metabolic networks (for some examples see references [34–38]), mainly based on protein homology and/or network topology. However they consider a small number of organisms or general metabolic maps of KEGG database. Also, many of these methods are in general difficult to compare with each other, as has been recently shown by Clack et al. [39].

In this work we used the alignment of linear enzymatic step sequences, similar to the previously described approaches [20, 40], where a general strategy for the systematic analysis of the metabolism in a multigenome scale was additionally implemented. The linear enzymatic alignment approach described here allows gaps using the NW algorithm, uses a random data comparison, and allows the identification of distant similarities like those observed between metabolic maps. To our knowledge, this is first time that these methods are used to compare systematically the metabolism of a well-studied and metabolic diverse clade. Therefore, our approach is able to capture directly the information contained in the individual metabolic networks of each organism.

Based on these comparisons, we detected a *core* of metabolic pathways associated with central carbohydrate metabolism, lipid, cell wall, and cofactors, and biosynthetic pathways. In contrast, variable maps are those associated with degradation pathways, except the glucose-related pathways and the TCA cycle. In addition, amino acid metabolism is an example of a pathway with multiple routes to yield similar compounds from different routes, characterized for alternative pathways.

In addition, two scenarios can be suggested to exemplify the growth of the metabolism. The first one, associated to the glycolysis, where a significant proportion of functional similarities from this pathway were observed in other metabolic pathways; suggesting the utilization of similar substrates/products processed by similar reactions in different metabolic maps. The second scenario is associated to the high number of hits for the IMP pathway associated to alignments within the same map, suggesting that this pathway possibly has increased its size by duplication and recruitment of its own enzymes and arising the possibility of major biochemical coupling restrictions for the recruitment of the enzymes in the IMP pathway. Therefore, the different patterns of ESS similarities of two ancient pathways suggest that the recruitment of catalytic activities in the metabolism is restricted by the metabolic context, being not a random phenomenon. Albeit our data suggest functional and, probably, evolutionary conservation of diverse catalytic steps, additional information must be considered to have a better approximation of metabolism evolution, such as gene transfer and gene loss, among other processes. For this reason, we do not exclude the possibility of diverse genetic phenomena, such as the continuum interchange of genetic material that diminishing the border between bacterial species, as it has been recently described in *E. coli* bacterial strains, where a small proportion of universal protein families [41] and a large proportion of specific families [42] have been found. In this regard, the functional conservation of metabolic steps was evaluated in a representative group of species selected with a genome similarity score of 0.7, as described by [43], capturing the general diversity of the *Gammaproteobacteria* metabolism.

Therefore, the method described here is able to identify alternative enzymes involved in similar metabolic processes, and although the conclusions can be restricted to the metabolism covered by *Gammaproteobacteria*, the method can be extended to any organism or clade for which there is metabolic information. Finally, we understand that the approach described here does not consider the effect of promiscuous enzymes, defined as those enzymes with more than two different E.C. numbers. However, previous analysis have described that around 10 % of the total enzymatic repertoire in bacterial and archaeal organisms corresponds to promiscuous enzymes [28, 44], suggesting that our results and conclusions are enough robust and can be little influenced by the multifunctional enzymes.

## Methods

### Selection of proteobacterial species

In this study we included the small-molecule metabolism of 40 different *Gammaproteobacteria* species. These organisms were selected from the 275 *Gammaproteobacteria* genomes included in the KEGG database as of June 2011 [3]. We chose non redundant genomes using the criteria described in reference [43], with a genome similarity score of 0.7, resulting in a set of 40 non redundant *Gammaproteobacteria* species. These organisms are representative of the division as it is shown in Additional file 2: Figure S4. Additional file 4: Table S2 contains the list of the organisms included in the analysis.

### Construction of ESS

**Fig. 6:**
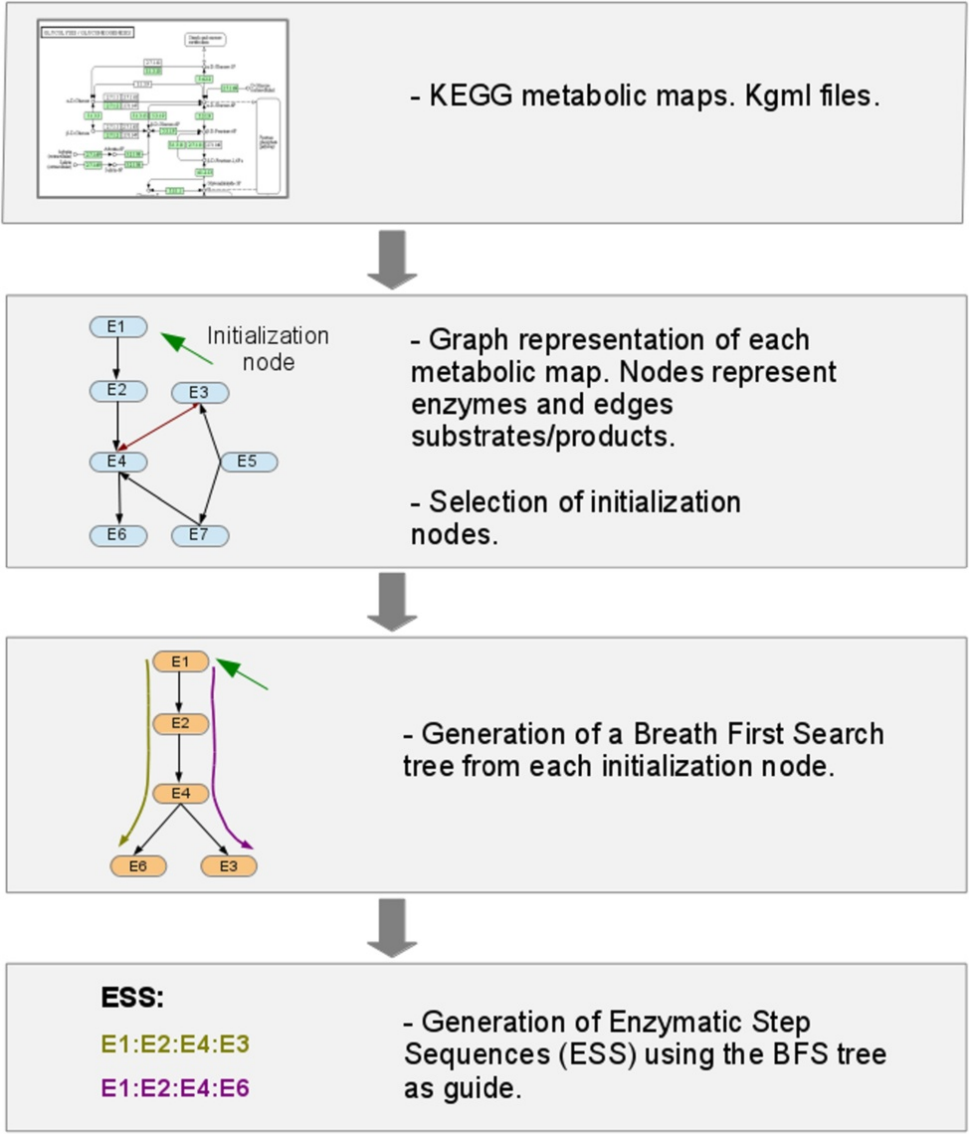
Strategy to generate the Enzymatic Step Sequences (ESS) from KEGG kgml files using the Breadth First Search (BFS) algorithm. First the kgml files are retrieved and directed graphs representations created. The nodes, represents genes or groups of genes (isozymes or complexes) that catalyze the reactions and the edges represent a compound that is a substrate from one reaction and a product for the next. Next a set of initialization nodes are selected using two criteria: a node which substrate is not catalyzed by another enzyme in the metabolic map; and, a node which substrate comes from another metabolic map and with two or less neighbors in the graph. Then, the initialization nodes are used as root for the construction of the BFS tree. Finally, the tree is used as guide to the construction of the nrESS. From each leaf (terminal node), the path is traced backwards until reach the root

We downloaded the KGML files (version 0.71) that describe the metabolic maps (pathways) of 40 *Gammaproteobacteria* in June 2011 from the KEGG database. Based on these metabolic maps, the ESS were constructed by using the BFS algorithm. In brief, a directed graphical representation of each metabolic map was created in which the nodes represented enzymes and the edges represented a shared substrate/product between two enzymes. This representation takes into account the reversibility of the reactions. In a posterior step, a group of BFS trees was generated for each metabolic map from a set of initialization nodes, used as roots. An initialization node was defined by two criteria: a node which substrate is not catalyzed by another enzyme in the metabolic map, and a node which substrate comes from another metabolic map and with two or fewer neighbors in the graph. These criteria represent the metabolic input for each pathway; the first criterion considers the substrates not created in the same pathway, and the second one considers the connections with other pathways. Each initialization node was used as a root for the construction of a BFS tree. Finally, each tree was used as a guide for the construction of the ESS. Thus, a BFS tree creates as many ESS as the number of branches it has. The graph representation of the metabolic maps and the BFS trees were generated using the Networkx Python module [45] (Fig. 6).

In a posterior stage, ESS were organized in a relational database. In this database, each EC number contained in a sequence was related to its corresponding protein(s), species, metabolic map. This database has a high degree of redundancy, because an ESS may be the same in different species. Thus, a nrESS dataset was constructed by filtering identical ESS and leaving only one representative. Each ESS in the nrESS dataset is linked to the original ESS. All analyses were conducted using the nrESS dataset and referring to the original data when necessary. The ESS and nrESS data are provided as supplementary material (Additional file 5: File S1).

### Comparison of nrESS by pairwise alignments

In order to identify the similarity of the nrESS, we implemented a pairwise alignment algorithm based on the Dynamic Programing Needleman and Wunsch (NW) algorithm as described in reference [46]. This algorithm works in a similar way as the classic tools to align nucleotide or amino acid sequences (Additional file 6: Text S1). We used an EC number weight matrix derived from an entropy-based evaluation function that evaluated the similarities between EC numbers. The weight matrix describes the similarity between the 136 different three levels EC numbers. The number 9.9.9 was used to describe an enzyme with no EC assigned and that was similar only to itself. The similarity between two EC numbers ranged from 0 to 1. Values close to 0 indicate similar EC numbers, and values close to 1 indicate different EC numbers. This matrix takes into account the hierarchy of the EC numbers, giving a value of 1 to all the EC pairs that are different in the first level of classification regardless of whether the second or third numbers are identical. Therefore, the NW algorithm uses the matrix to construct an alignment that minimizes the global score. Finally, the alignment is evaluated by using the normalized entropy-based function. The *score* obtained with such an evaluation function also ranges from 0 to 1, where 0 indicates similar nrESS and 1 dissimilar nrESS. To analyze in more detail the similarities of the low part of the glycolysis and the IMP pathways, their ESS were compared against the nrESS. Examples of nrESS alignments are shown in Additional file 2: Figure S5.

### Statistically significant ESS alignments

To determine the statistical significance of the nrESS alignments, we compared the alignment scores of the real database against the scores from10 different random databases. These random sequences were constructed by shuffling the EC number content of the entire database, maintaining the nrESS length and EC composition of the original sequences. Each random database was submitted to the same all-versus-all alignment approach used for the real data, and the distribution of alignment scores considered the mean ± SD. The threshold considered statistically significant corresponded to a score of ≤0.3, i.e., that point with higher dispersion of the real data relative to the mean random databases scores and where the loss of nrESS due to extreme dissimilarity was less than 1 %, i.e. this threshold includes the 99 % of the nrESS.

### Functional conservation of enzymatic steps in metabolic maps

We used the information provided by the nrESS pairwise alignments to identify the *functionally conserved* enzymatic steps in *Gammaproteobacteria* for each metabolic map. Two nrESS were considered conserved if their alignment scores were below or equal to 0.3 and if, in conjunction, they were present in more than 75 % of the organisms. This criterion was employed because we assumed that a pair of conserved ESS would be shared by at least all of the species with genomes greater than 2000 ORFs, i.e., 30 of the 40 *Gammaproteobacteria* organisms. From the ESS that fulfilled this criterion, we selected those that corresponded to the same metabolic map. This subset of sequences was named the Metabolic Map Functional Conserved Dataset (MMFCD). To identify the conserved ESS, the aligned identical EC numbers from each alignment were mapped in the corresponding position in KEGG metabolic maps.

### Clustering of similar metabolic maps

In order to identify the functional similarities among metabolic maps, we selected a subset of nrESS pairwise alignments with score values of ≤0.3. These alignments were used to construct a similarity matrix where each cell corresponded to the count of the alignments shared by each pair of metabolic maps. The rows representing the metabolic maps were normalized by the total alignments in each row. The matrix was used as input to a hierarchical clustering analysis with the program MeV4 (http://www.tm4.org/mev.html). The similarity between maps was calculated with the Spearman’s rank correlation, and elements were clustered with the average method. A cutoff of 0.46 of the total length of the dendogram was used to classify the metabolic maps into groups and is displayed with the E.T.E. 2 Python toolkit [47].

## Additional files

Additional file 1: Table S1. Statistics of construction of ESS per metabolic map. Number of reactions, genes, and ESS produced for each metabolic map per organism. (TAB 230 kb) Additional file 2:
**Figure S1.** Number of ESS generated by metabolic map. In X-axis, denotes the number of enzymes per metabolic map; Y-axis corresponds to the number of ESS. Each point represents one metabolic map. The data were adjusted to a linear model. **Figure S2.** Alignment scores of the nrESS database comparisons. The distribution resembles an extreme value *Gumbel* distribution skewed to the right. Scores are close to 1 and represent alignments of dissimilar sequences. In counterpart, scores close to 0 correspond to alignments between similar sequences. **Figure S3.** Proportion of nrESS included in at least one pairwise alignment as function of the alignment score. X-axis represents the threshold at different values, whereas the Y-axis shows the proportion of nrESS included. The number of excluded nrESS decreases as the alignment score increases. The proportion of included nrESS of the real data is compared with the same proportion of 10 random databases. In the last case, the proportion of included ESS decreases abruptly at scores close to 0. **Figure S4.** *Gammaproteobacteria* coverage of the organisms used in this study. The organisms used in this study are marked as circles. The organisms listed in the upper right corner are not represented in the phylogenetic tree. The tree was taken and modified from [14]. **Figure S5.** Pairwise ESS alignments. A NW algorithm was used in these examples. The aligned pairs of ESS and their corresponding scores are indicated. Gaps in the alignment are indicated by dashes (-.-.-). Significant scores are those with scores ≤ 0.3. (PDF 4362 kb) Additional file 3: File S2. KEGG weblinks with the functional conservation of metabolic maps in *Gammaproteobacteria. (TXT 56 kb)*
Additional file 4: Table S2. Organisms considered in this study. Genomic and metabolic information associated to each organism analyzed in this work. (DOCX 31 kb) Additional file 5: File S1. Enzymatic Step Sequence database. (ZIP 1300 kb) Additional file 6: Text S1. Description of the NW ESS alignment functions. (DOC 72 kb)
