# Supplementary material for: The alignment of enzymatic steps reveals similar metabolic pathways and probable recruitment events in Gammaproteobacteria
Source: BMC Genomics. 2015 Nov 17;16:957. doi: 10.1186/s12864-015-2113-0 (PMC4647829; doi:10.1186/s12864-015-2113-0)
Supplement: Additional file 2: — Figure S1. Number of ESS generated by metabolic map. In X-axis, denotes the number of enzymes per metabolic map; Y-axis corresponds to the number of ESS. Each point represents one metabolic map. The data were adjusted to a linear model. Figure S2. Alignment scores of the nrESS database comparisons. The distribution resembles an extreme value Gumbel distribution skewed to the right. Scores are close to 1 and represent alignments of dissimilar sequences. In counterpart, scores close to 0 correspond to alignments between similar sequences. Figure S3. Proportion of nrESS included in at least one pairwise alignment as function of the alignment score. X-axis represents the threshold at different values, whereas the Y-axis shows the proportion of nrESS included. The number of excluded nrESS decreases as the alignment score increases. The proportion of included nrESS of the real data is compared with the same proportion of 10 random databases. In the last case, the proportion of included ESS decreases abruptly at scores close to 0. Figure S4. Gammaproteobacteria coverage of the organisms used in this study. The organisms used in this study are marked as circles. The organisms listed in the upper right corner are not represented in the phylogenetic tree. The tree was taken and modified from [14]. Figure S5. Pairwise ESS alignments. A NW algorithm was used in these examples. The aligned pairs of ESS and their corresponding scores are indicated. Gaps in the alignment are indicated by dashes (-.-.-). Significant scores are those with scores ≤ 0.3. (PDF 4362 kb) [file 12864_2015_2113_MOESM2_ESM.pdf]

Supporting information.

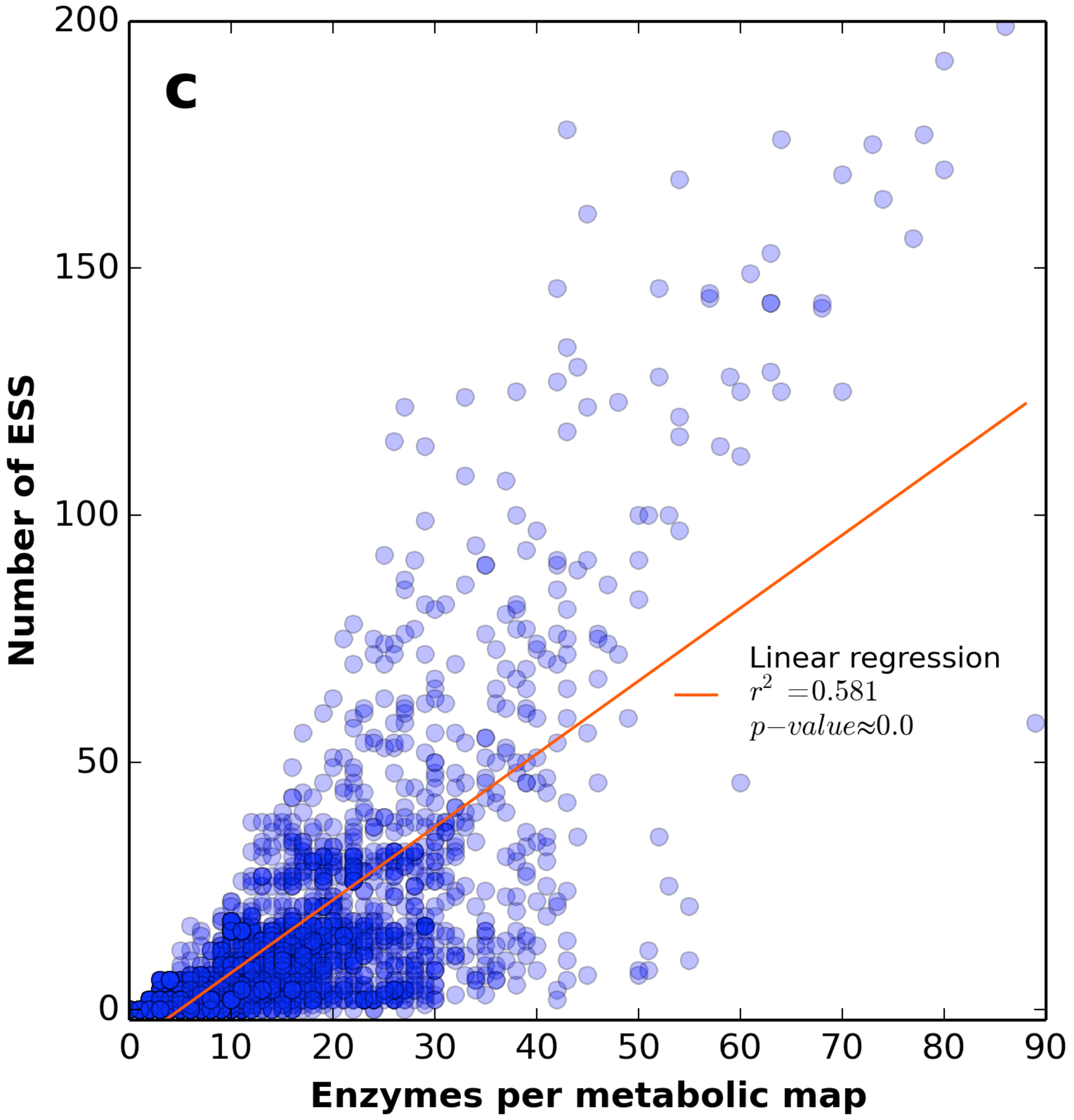

Figure S1. Number of ESS generated by metabolic map. In X-axis, denotes the number of enzymes per metabolic map; Y-axis corresponds to the number of ESS. Each point represents one metabolic map. The data were adjusted to a linear model.

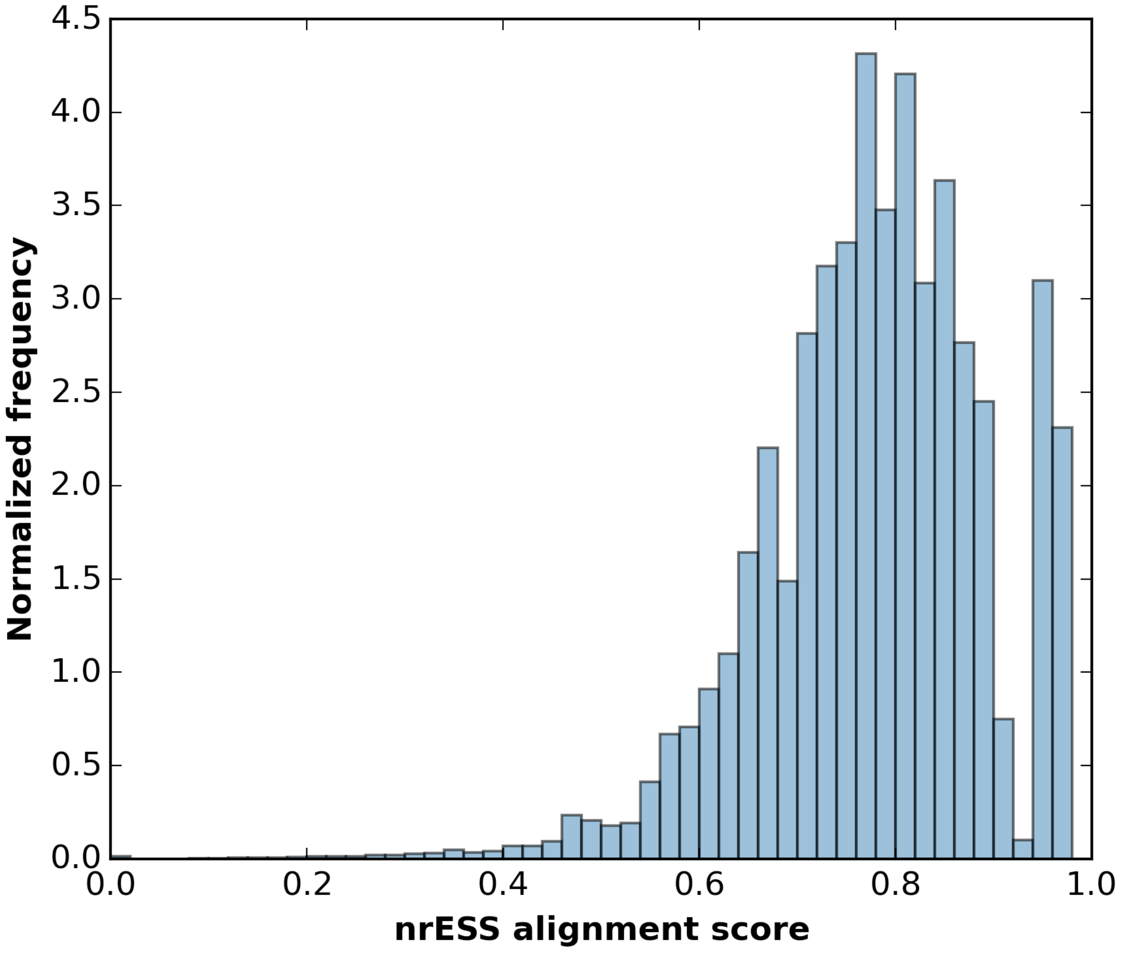

Figure S2. Alignment scores of the nrESS database comparisons. The distribution resembles an extreme value *Gumbel* distribution skewed to the right. Scores are close to 1 and represent alignments of dissimilar sequences. In counterpart, scores close to 0 correspond to alignments between similar sequences.

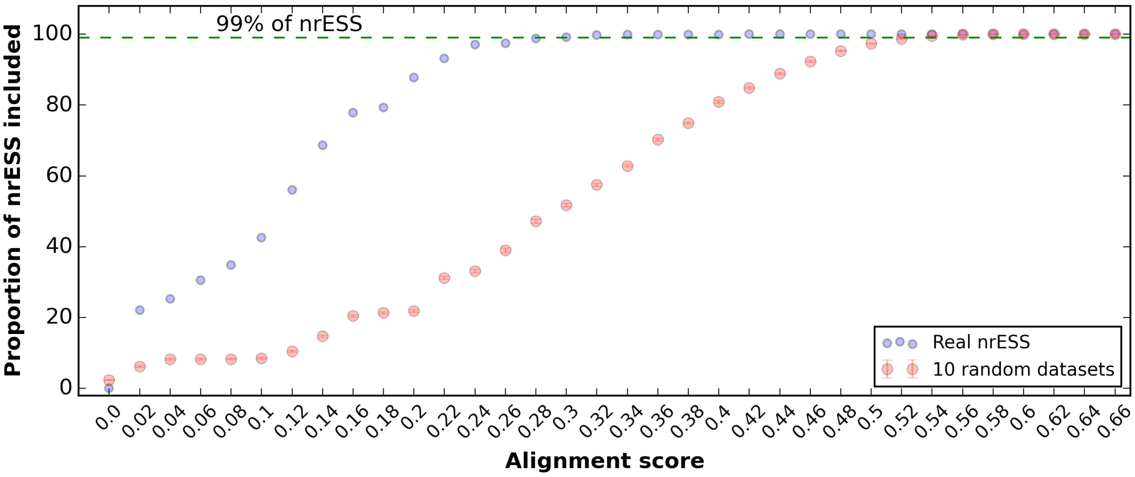

Figure S3. Proportion of nrESS included in at least one pairwise alignment as function of the alignment score. X-axis represents the threshold at different values, whereas the Y-axis shows the proportion of nrESS included. The number of excluded nrESS decreases as the alignment score increases. The proportion of included nrESS of the real data is compared with the same proportion of 10 random databases. In the last case, the proportion of included ESS decreases abruptly at scores close to 0.

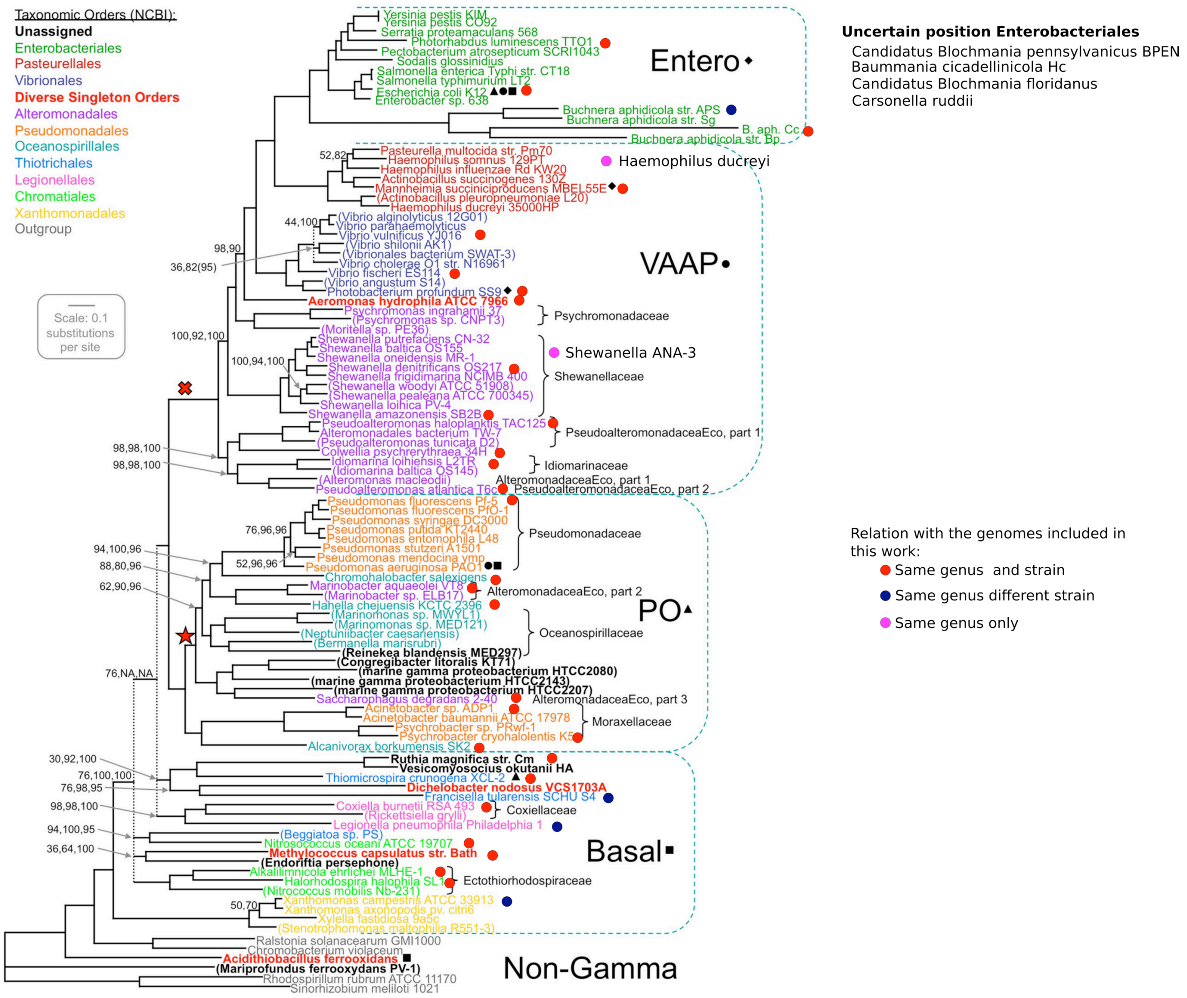

Figure S4. ***Gammaproteobacteria* coverage of the organisms used in this study.** The organisms used in this study are marked as circles. The organisms listed in the upper right corner are not represented in the phylogenetic tree. The tree was taken and modified from [14].

nrid	1	2	3	4	5	6	7	8	9	10	11	12	13
7454	-.-.-	6.3.4	2.1.2	6.3.5	6.3.3	6.3.4	5.4.99	6.3.2	4.3.2	2.4.2	2.7.1	-.-.-	-.-.-
2528	2.4.2	6.3.4	2.1.2	6.3.5	6.3.3	6.3.4	5.4.99	6.3.2	4.3.2	2.7.4	2.7.1	2.7.6	3.6.1

score = 0.25015

nrid	1	2	3	4
7883	6.4.1	2.3.1	2.7.2	1.2.1
7882	6.4.1	2.3.1	2.7.2	-.-.-

score = 0.2375

nrid	1	2	3	4	5	6	7	8	9	10
1388	1.5.99	1.2.1	2.6.1	4.1.1	3.5.1	2.1.3	2.7.2	1.4.1	3.5.1	1.2.1
1383	1.5.99	1.2.1	2.6.1	4.1.1	3.5.1	2.1.3	2.7.2	1.4.1	1.5.1	1.5.1

score = 0.13519

nrid	1	2	3	4	5	6	7
3614	2.7.2	1.2.1	1.1.1	2.3.1	2.5.1	2.3.1	2.5.1
1748	2.1.3	4.3.1	1.5.99	-.-.-	1.5.1	2.3.1	-.-.-

score = 0.68269

nrid	1	2	3
1653	-.-.-	2.1.3	3.5.1
1542	2.1.1	2.1.1	1.3.1

score = 0.64551

nrid	1	2	3	4	5
6600	4.3.2	3.5.3	4.3.1	1.5.1	2.7.2
4214	3.1.1	3.1.4	4.3.1	-.-.-	-.-.-

score = 0.65038

nrid	1	2	3	4	5	6
3857	-.-.-	-.-.-	-.-.-	2.7.4	3.6.1	-.-.-
1288	1.5.1	3.5.1	1.2.1	2.6.1	3.5.3	2.3.1

score = 0.76731

nrid	1	2	3
6781	4.4.1	4.3.1	-.-.-
185	1.1.1	2.7.1	1.1.1

score = 0.95

nrid	1	2	3	4	5	6	7	8
3739	2.7.2	1.4.1	2.3.1	2.7.2	1.2.1	2.6.1	3.5.1	2.1.3
2760	2.5.1	-.-.-	-.-.-	4.2.1	1.3.1	-.-.-	-.-.-	-.-.-

score = 0.82548

Figure S5. Pairwise ESS alignments. A NW algorithm was used in these examples. The aligned pairs of ESS and their corresponding scores are indicated. Gaps in the alignment are indicated by dashes (-.-.-). Significant scores are those with scores ≤ 0.3.
