# Supplementary material for: The alignment of enzymatic steps reveals similar metabolic pathways and probable recruitment events in Gammaproteobacteria
Source: BMC Genomics. 2015 Nov 17;16:957. doi: 10.1186/s12864-015-2113-0 (PMC4647829; doi:10.1186/s12864-015-2113-0)
Supplement: Additional file 4: Table S2. — Organisms considered in this study. Genomic and metabolic information associated to each organism analyzed in this work. (DOCX 31 kb) [file 12864_2015_2113_MOESM4_ESM.docx]

**Table S2. Organisms considered in this study sorted by number of ORFs.** Nomenclature is as follows: KID, KEGG ID; Organisms; Number of ORFs; Maps, number of KEGG metabolic maps; ECs, Number of distinct EC numbers (4 levels of classification); O-ECs, number of ORFs assigned to at least one EC number; Biotic Relationship (BR), S, Symbiotic or Free living; Energy Source (ES): H, Heterotroph, A, Autotroph, Ch, Chemoheterotroph, Ca, Chemoautotroph, Cl, Chemolitotroph, Co, Chemoorganotroph, Pp, Phototroph, P, Photosynthetic, M, Methanotroph. The BR, ES and Metabolism information were obtained from Integrated Microbial Genomes (IMG) database.

| **KID** | **Organism** | **ORFs** | **Maps** | **ECs** | **O-ECs** | **BR** | **ES** | **Metabolism** |
| --- | --- | --- | --- | --- | --- | --- | --- | --- |
| hch | *Hahella chejuensis KCTC2396* | 6778 | 81 | 734 | 1172 | F | H | Marine red-pigmented; not pathogen |
| pfl | *Pseudomonas protegens Pf-5* | 6139 | 84 | 822 | 1432 | F | H | Inhabit soil, plants, and water surfaces. It is an organism that causes discoloration and pitting of mushroom caps |
| pr | *Photobacterium profundum SS9* | 5489 | 77 | 758 | 1254 | F |  | Barophilic and can survive under high pressures |
| cps | *Colwellia psychrerythraea 34H* | 4910 | 79 | 684 | 1075 | F |  | Obligate psychrophile |
| plu | *Photorhabdus luminescens* | 4683 | 78 | 732 | 1086 | S |  | Symbiotic relationship with soil entomopathogenic nematodes |
| xca | *Xanthomona campestris vesicatoria 85-10* | 4469 | 85 | 718 | 1078 | F |  | Pathogen. Bacterial leaf spot |
| vvu | *Vibrio vulnificus YJ016* | 4433 | 78 | 717 | 1096 | F | H | Halophile; virulent bacterium associated with about 95 percent of all seafood related deaths |
| pat | *Pseudoalteromona satlantica T6c* | 4281 | 85 | 719 | 1063 | F | Co | Cause shell disease-infected edible crabs |
| maq | *Marinobacter aquaeolei VT8* | 4272 | 79 | 640 | 988 | F | H | Iron oxidizer; hydrocarbon degrading |
| eco | *Escherichia coli K12* | 4150 | 81 | 859 | 1369 | F |  | Facultative anaerobe |
| aha | *Aeromonas hydrophila ATCC7966* | 4121 | 79 | 751 | 1218 | F | H | Found in a variety of aquatic environments worldwide, including bottled water, chlorinated water, well water, and heavily polluted waters |
| shm | *Shewanella ANA-3* | 4014 | 78 | 678 | 1025 | F | H | Salinity tolerance |
| sde | *Saccharophagus degradans 2-40* | 4007 | 75 | 604 | 876 | F |  | Ethanol production, Cellulose degrader |
| vfi | *Vibrio fischeri ES114* | 3817 | 77 | 694 | 1120 | S | H | Predominantly in symbiosis with various marine animals, such as the bobtail squid |
| sdn | *Shewanella denitrificans OS217* | 3754 | 80 | 644 | 937 | F | H | Denitrifying |
| saz | *Shewanella amazonensis SB2B* | 3645 | 79 | 690 | 1022 | F | H | Metal reducer |
| pin | *Psychromonas ingrahamii 37* | 3548 | 75 | 677 | 997 | F |  | Psychrophilic bacteria |
| pha | *Pseudoalteromonas haloplanktis TAC125* | 3489 | 82 | 672 | 1002 | F |  | Isolated from the Antarctic coastal sea sample water and is found to grow only in marine environment at ~20ºC |
| aci | *Acinetobacter ADP1* | 3308 | 84 | 648 | 1008 | F | H, Ch | Aerobic negative bacilli. |
| csa | *Chromohalobacter salexigens DSM3043* | 3298 | 79 | 679 | 971 | F | Co | Aerobic chemoorganotroph |
| noc | *Nitrosococcus oceani ATCC19707* | 3018 | 77 | 599 | 851 | F | Cl, A | Nitrifying, Ammonia-oxidizer |
| mca | *Methylococcus capsulatus Bath* | 2956 | 72 | 581 | 844 | F | M | Methylococcus capsulatus |
| lpn | *Legionella pneumophila Paris* | 2943 | 79 | 550 | 788 | F | Co | Aerobic, pleomorphic, flagellated |
| aeh | *Alakalilimnicola ehrlichei MLHE-1* | 2865 | 77 | 584 | 850 | F | Ca, Cl | Arsenite oxidizer |
| abo | *Alcanivorax borkumensis SK2* | 2755 | 81 | 606 | 931 | F | O | Hydrocarbon degrading |
| ilo | *Idiomarina loihiensis L2TR* | 2628 | 73 | 557 | 752 | F |  | Survey of bacterial diversity along deep sea hydrothermal vents |
| pcr | *Psychrobacter cryohalolentis K5* | 2511 | 80 | 600 | 820 | F |  | It was isolated from a cryopeg within permafrost in the Kolyma lowland, Siberia, Russia. This strain is able to reproduce at -10 °C |
| hha | *Halorhodospira halophila SL1* | 2407 | 67 | 567 | 753 | F | Pp, P | Extremely halophilic purple bacterium |
| msu | *Mannheimia succiniciproducens MBEL55E* | 2369 | 69 | 563 | 780 | F |  | Succinic acid production |
| tcx | *Thiomicrospira crunogena XCL-2* | 2196 | 64 | 500 | 695 | F | Ca | Sulfur oxidizer, Desulfurylates coal, Carbon dioxide fixation |
| cbu | *Coxiella burnetii* | 1847 | 68 | 414 | 530 | S |  | Obligate intracellular bacterial pathogen, and is the causative agent of Q fever |
| hdu | *Haemophilus ducreyi 35000HP* | 1717 | 56 | 409 | 546 | F |  | Coccobacillus causing the sexually transmitted disease chancroid |
| ftu | *Francisella tularensis holarctica* | 1604 | 71 | 431 | 581 | F |  | Pathogenic species and the causative agent of tularemia |
| rma | *Candidatus Ruthia magnifica Cm* | 976 | 57 | 396 | 488 | S | Ca | Sulfur oxidizer, Nitrogen fixation |
| bpn | *Candidatus Blochmania pennsylvanicus BPEN* | 610 | 52 | 301 | 365 | F |  | Endosymbiont of carpenter ants |
| bci | *Baummania cicadellinicola Hc* | 595 | 44 | 298 | 346 | F |  | Symbiotic |
| bfl | *Candidatus Blochmania floridanus* | 583 | 52 | 288 | 351 | F |  | Endosymbiont of carpenter ants |
| buc | *Buchnera aphidicola* | 574 | 45 | 278 | 331 | S |  | Symbiotic relationship |
| bcc | *Buchnera aphidicola Cc* | 362 | 31 | 161 | 195 | S |  | Symbiotic relationship |
| crp | *Carsonella ruddii* | 182 | 19 | 69 | 78 | S |  | Obligate endosymbiont to the insects psyllids |
