# Supplementary material for: The alignment of enzymatic steps reveals similar metabolic pathways and probable recruitment events in Gammaproteobacteria
Source: BMC Genomics. 2015 Nov 17;16:957. doi: 10.1186/s12864-015-2113-0 (PMC4647829; doi:10.1186/s12864-015-2113-0)
Supplement: Additional file 6: Text S1. — Description of the NW ESS alignment functions. (DOC 72 kb) [file 12864_2015_2113_MOESM6_ESM.doc]

**SUPPORTING TEXT 1**

**Dynamic programming Needleman and Wunsch (NW) algorithm for alignment of Enzymatic Step Sequences (ESS).**

In order to calculate the similarity between two ESS, we implemented a Dynamic Programming (DP) method based in the NW algorithm as it is proposed in [1]. Here, we defined an ESS as a sequence of biochemically coupled EC numbers.

The similarity between 2 three level EC numbers (*l_1_.l_2_.l_3_*), *ECS(EC_1_,EC_2_)*, was evaluated according the following normalized equation:

$$ECS(EC_{1},EC_{2})=\frac{w_{1}H_{1}+w_{2}H_{2}+w_{3}H_{3}}{w_{1}w_{2}w_{3}}$$

Where *w_1_, w_2_, w_3_* correspond to the weight factors for each EC number classification level and *H_1_, H_2_, H_3_* to the entropy measured for each aligned level. The weight factors used were 15, 10 and 1 for the first, second and third classification levels respectively. These factors were selected empirically based on the quality of the alignments generated. The entropy
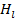
for each classification level, *l*, was calculated according the following equation based in information theory [2]:

$$H_{l}=\sum_{i=1}^{s} p_{i}\log_{2}(p_{i})$$

Where *s* is the number of classifications in the aligned level *l*and *p_i_* is the probability to find the classification *i* in the level *l*. The entropy can be simplified for the pairwise alignment case as follows: If the classifications of both EC numbers in *l* are equal, then *H_l_* = 0, if they are different then *H_l_ = 1*. Therefore, when the entropy value for two EC numbers is high, the similarity (information) is small. In counterpart, when the entropy value is low, the similarity is large. For this reason, the DP algorithm tries to minimize the scoring of the alignment instead to maximize it, as it is the case for the nucleotide and amino acid alignment algorithms.

The EC number similarity equation was created intended to be useful for the evaluation of multiple ESS alignments, and our unpublished results suggest that this measure is qualitatively more efficient for multiple sequence alignment than the classical *sum of pairs* approach in a genetic algorithm context.

An EC number similarity matrix, *S*, was constructed using this measure. The matrix represents the pairwise comparison of 135 different 3 levels of the EC numbers. The number 9.9.9 was used to describe enzymes with no EC assigned and it is similar only to itself. This matrix takes into account the hierarchy of the EC numbers, giving a value of 1 to all the EC pairs that are different in the first level of classification regardless of whether the second or third numbers are identical.

To perform the NW algorithm of *ESS_1_* and *ESS_2_* a DP matrix, *M*, with shape $(n+1)*(m+1)$ is constructed, where *n* is the length of *ESS_1_* and *m* is the length of *ESS_2_*.Thematrix *M* is filled according the following rule. For each cell *M_i,j_*:

$$M_{i,j}=\min\left\{\begin{matrix} M_{i-1,j-1}+S_{EC_{i}EC_{j}} \\ M_{i,j-1}+gap \\ M_{i-1,j}+gap \end{matrix}\right.$$

Where $S_{EC_{i}EC_{j}}$ is the similarity assigned in the matrix *S* between the EC numbers *EC_i_* and *EC_j_*. The *gap* penalization value is set to 1, equal to the maximum dissimilarity between two EC numbers. For details about NW algorithm refers to [1].

**Alignment scoring function**

After the application of the DP algorithm, the alignment is evaluated using a normalized function. This function also facilitates the evaluation of future multiple ESS alignments. The *score* is calculated:

$$score=0.95(H)+0.05(GP)$$

Where *H* is the measure of the mean homogeneity (entropy) in the aligned columns and *GP* is the penalization for the gaps.

The homogeneity is measured by the equation:

$$H=\frac{\sum_{i=1}^{n} S_{EC_{i1}EC_{i2}}}{n}$$

Where *n* is the number of aligned EC numbers in the alignment and$S_{EC_{i1}EC_{i2}}$ is the similarity value in the matrix *S* of the two EC numbers aligned in the position *i*. The gap penalization is measured by the equation:

$$GP=\frac{\sum_{i=1}^{ns} \frac{GB_{i}}{TG_{i}}}{ns}$$

Where *GB_i_* is the number of gap blocks in the ESS *i*, *TG_i_* is the total number of gap characters in the ESS *i* (the gap character is “-.-.-“) and *ns* is the number of ESS (2 for a pairwise alignment). This equation evaluates the concentration of the gaps. The numerator became smaller, as the number of gap blocks increases. As in the alignment of nucleotide or amino acid sequences, it is more parsimonious one large gap, than many short gaps. The gaps at the end of the ESS are not included in the count of gaps or in the measurement of the homogeneity of the alignment.

References

1. Kinser J (2008) Python For Bioinformatics. Jones & Bartlett Publishers. Available: http://www.amazon.com/dp/0763751863.

2. Shannon C (1948) A mathematical theory of communication. Bell Syst Tech J 27: 379–423. Available: http://dl.acm.org/citation.cfm?id=584093.
